# Efficacy and safety of Sacituzumab govitecan in solid tumors: a systematic review and meta-analysis

**DOI:** 10.3389/fonc.2025.1624386

**Published:** 2025-06-20

**Authors:** Yaping Zhang, Jian Chen, Xiaoyan Wang, Hui Wang, Xiaoli Chen, Jianfeng Hong, Hongming Fang

**Affiliations:** ^1^ Department of Oncology, Affliated Xiaoshan Hospital, Hangzhou Normal University, Hangzhou, China; ^2^ Department of GCP, Affiliated Xiaoshan Hospital, Hangzhou Normal University, Hangzhou, China

**Keywords:** Sacituzumab govitecan, solid tumors, breast cancer, lung cancer, urothelial cancer

## Abstract

**Background:**

Sacituzumab govitecan (SG) is an antibody-drug conjugate (ADC) that targets trophoblast cell-surface antigen 2 and is conjugated to SN-38, a potent topoisomerase I inhibitor. SG has demonstrated promising activity against various solid tumors. However, comprehensive evaluations of its efficacy and safety remain limited, and outcomes across studies have shown inconsistency. This systematic review and meta-analysis aimed to assess the therapeutic efficacy and associated adverse events (AEs) of SG in the treatment of solid tumors.

**Methods:**

A systematic search of PubMed, Embase, and the Cochrane Library was conducted to identify randomized controlled trials (RCTs) and single-arm studies published up to February 14, 2025. The primary outcomes included overall response rate (ORR), disease control rate (DCR), progression-free survival (PFS), overall survival (OS), and treatment-related AEs.

**Results:**

Five RCTs comparing SG with chemotherapy were included in the analysis. SG significantly improved OS (risk ratio [RR] = 0.720; 95% confidence interval [CI]: 0.587–0.882; P = 0.002), PFS (RR = 0.682; 95% CI: 0.516–0.901; P = 0.007), and DCR (RR = 1.286; 95% CI: 1.034–1.599; P = 0.024). However, higher incidences of neutropenia, leukopenia, diarrhea, and anemia were observed in the SG group. In the single-arm meta-analysis, 16 cohorts from five trials were included. The pooled ORR was 21% (95% CI: 16%–27%), DCR was 62% (95% CI: 56%–69%), and the clinical benefit rate was 33% (95% CI: 26%–39%). The median pooled PFS, duration of response, and OS were 4.41 months (95% CI: 3.61–5.22 months), 7.40 months (95% CI: 5.99–8.82 months), and 10.29 months (95% CI: 7.75–12.83 months), respectively.

**Conclusion:**

Although SG demonstrates superior OS, PFS, and DCR compared to chemotherapy in patients with solid tumors, this benefit is accompanied by an increased risk of specific adverse events. Subgroup analyses indicate that SG confers a more substantial clinical benefit in breast and urothelial cancers than in other tumor types.

## 1 Introduction

Cytotoxic chemotherapies have long been the cornerstone of cancer treatment (1). However, their effectiveness is constrained by a narrow therapeutic index, resulting in significant off-target toxicity due to non-specific drug distribution (2, 3). To overcome these limitations and improve clinical outcomes, molecularly targeted therapies—particularly antibody-drug conjugates (ADCs)—have emerged as a promising alternative.

ADCs combine the target specificity of monoclonal antibodies with the cytotoxic potency of chemotherapeutic agents, linked via a chemical linker. This design allows for the selective delivery of cytotoxic agents to tumor-specific antigens, thereby enhancing therapeutic efficacy while reducing systemic toxicity—advantages that conventional chemotherapy cannot offer (4). Several ADCs have received approval from the United States Food and Drug Administration (FDA) for the treatment of cancer (5, 6).

Among these, sacituzumab govitecan (SG) has demonstrated notable efficacy in breast, lung, urothelial, gastric, and other solid tumors (7–10). SG targets trophoblast cell-surface antigen 2 (Trop-2), a protein frequently overexpressed in aggressive tumors and associated with poor prognosis (10). It delivers SN-38, a potent topoisomerase I inhibitor, directly to Trop-2–expressing cancer cells, thereby minimizing damage to normal tissues (11, 12). SG was the first Trop-2–targeting ADC to receive regulatory approval. In April 2020, the FDA granted accelerated approval for SG in patients with metastatic triple-negative breast cancer who had received at least two prior systemic therapies, based on phase I/II trials demonstrating a high overall response rate (ORR) and durable clinical benefit (5, 13). A second accelerated approval followed in April 2021 for patients with locally advanced or metastatic urothelial carcinoma who had progressed after platinum-based chemotherapy and PD-1/PD-L1 inhibitor therapy (14). Despite these important milestones, SG’s clinical profile remains under evaluation. Some studies have reported higher incidences of grade ≥3 treatment-emergent adverse events (TEAEs) compared to conventional chemotherapy (15). Therefore, a thorough assessment of SG’s efficacy and safety is warranted. This study presents a meta-analysis of clinical trials evaluating SG across various solid tumors. Key outcomes include ORR, disease control rate (DCR), progression-free survival (PFS), overall survival (OS), and adverse events (AEs), with the aim of providing evidence-based guidance to inform clinical decision-making.

## 2 Methods

### 2.1 Search strategy

A systematic review and meta-analysis were conducted to evaluate the efficacy and safety of SG in solid tumors. Comprehensive literature searches were performed in PubMed, Embase, and the Cochrane Library for English-language publications available up to February 14, 2025. No restrictions were applied regarding the year of publication. Case reports, case series, and review articles were excluded. The search strategy included the following terms: “Sacituzumab Govitecan,” “Sacituzumab Govitecan-hziy,” “IMMU-132,” “Trodelvy,” “GW786034,” “cancer,” and “tumor.”

### 2.2 Inclusion and exclusion criteria

Studies were included if they met the following criteria: (1) prospective phase 1–3 clinical trials evaluating SG in adults with histologically confirmed solid tumors; (2) randomized controlled trials (RCTs) comparing SG with chemotherapy; (3) single-arm trials assessing SG as monotherapy; (4) reporting of at least two of the following outcomes, including OS, PFS, ORR, and DCR; (5) reporting of grade ≥3 TEAEs as secondary outcomes; (6) when multiple reports were available from the same study, the most recent or comprehensive report was selected; and (7) availability of full-text articles published in English. Exclusion criteria were as follows: (1) retrospective studies; (2) *in vitro* or *in vivo* preclinical research; (3) case reports or case series, or studies with fewer than 10 participants; (4) non-English publications; (5) studies evaluating SG in combination with other therapeutic agents; and (6) studies with insufficient data or lack of full-text access.

### 2.3 Literature screening and data extraction

Titles and abstracts were independently screened by two investigators (Y.P. Zhang and J. Chen) based on predefined inclusion and exclusion criteria. Discrepancies were resolved through discussion, and any unresolved cases were adjudicated by a third reviewer (H.M. Fang) through majority consensus. When abstracts lacked sufficient information to determine eligibility or did not contain relevant data, full-text articles were reviewed.

Following study selection, data were extracted on key study characteristics, including the first author, year of publication, sample size, patient age, tumor type, prior treatments, study design, and primary outcomes. Extracted outcomes included OS, PFS, ORR, DCR, and TEAEs of grade ≥3. Data were obtained from both the main text and **Supplementary Materials**.

### 2.4 Quality assessment

The methodological quality of the included RCTs was independently assessed by two reviewers (X.Y. Wang and H. Wang). Any disagreements were resolved through consensus or, if necessary, by consultation with a third reviewer (X.L. Chen). The assessment was conducted using the Cochrane Risk of Bias Tool, as outlined in the Cochrane Handbook for Systematic Reviews of Interventions (16). Risk of bias was evaluated across the following domains: random sequence generation, allocation concealment, blinding of participants and personnel, blinding of outcome assessment, incomplete outcome data, selective reporting, and other potential sources of bias. Each domain was classified as having a low, high, or unclear risk of bias. A visual summary of the risk of bias was generated using Review Manager (RevMan) version 5.4 (The Nordic Cochrane Centre, The Cochrane Collaboration, Copenhagen, 2020).

### 2.5 Statistical analysis

Statistical analyses were conducted using Stata version 12.0 (StataCorp, College Station, TX, USA). Dichotomous outcomes, including ORR and DCR, were analyzed using relative risks (RRs), while hazard ratios (HRs) with 95% confidence intervals (CIs) were extracted for OS and PFS. Pooled HRs were estimated using either fixed-effects or random-effects models, depending on the degree of heterogeneity among studies. Heterogeneity was evaluated using the I^2^ statistic and the Chi-squared test, with I^2^ ≥ 50% considered indicative of substantial heterogeneity. A random-effects model was employed when significant heterogeneity was detected (I^2^ > 50% or P < 0.05); otherwise, a fixed-effects model was applied (17). Publication bias was assessed using Begg’s test and visual inspection of funnel plots. A two-tailed P value < 0.05 was considered statistically significant (18).

## 3 Results

### 3.1 Literature selection

**Figure 1:**
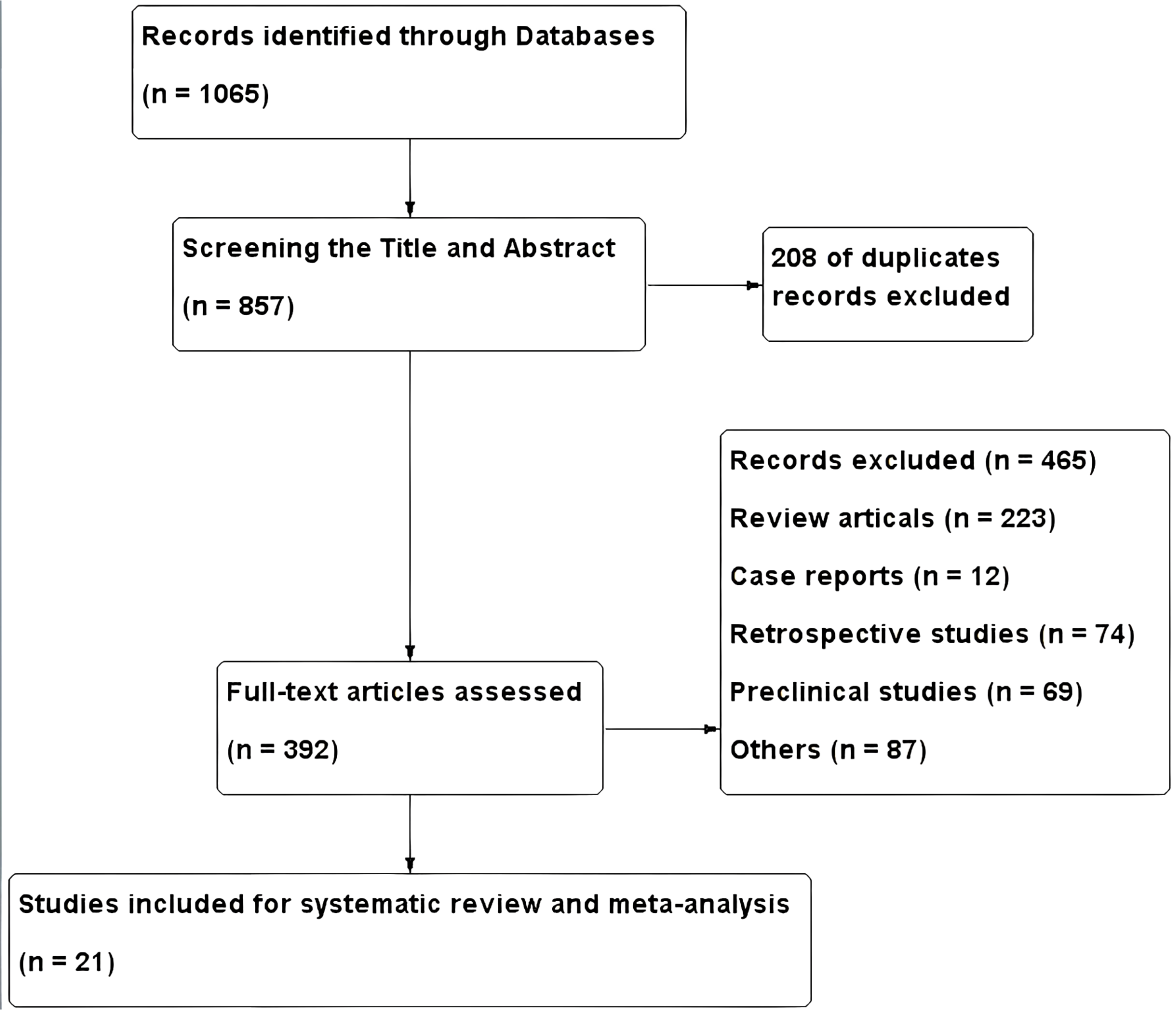
The flowchart of the study selection process for the meta-analysis.

Using the predefined search strategy, a total of 1,712 studies published between March 2015 and February 14, 2025, were identified. After title and abstract screening, 116 full-text articles were assessed for eligibility. Ten studies met the inclusion criteria: five RCTs were included in the comparative meta-analysis, and five phase I–II trials were included in the single-arm analysis. The study selection process is illustrated in **Figure 1**.

### 3.2 RCTs

#### 3.2.1 General characteristics of included RCTs

This meta-analysis included five phase III RCTs comprising a total of 2,717 patients—1,359 treated with SG and 1,358 with chemotherapy (7–9, 15, 19). The studies focused on three tumor types: non-small cell lung cancer (NSCLC) (8), urothelial carcinoma (UC) (9), and breast cancer (BC), including both triple-negative and hormone receptor-positive/human epidermal growth factor receptor 2-negative (HR+/HER2-) subtypes (7, 15, 19). In all trials, SG was compared with the physician’s choice of single-agent chemotherapy. The EVOKE-01 trial used docetaxel as the comparator (8), while the remaining studies included eribulin, vinorelbine, capecitabine, gemcitabine, or vinflunine (7, 9, 15, 19). Key study characteristics and main outcomes are presented separately in **Tables 1** and **2**.

**Table 1:** Characteristics of the five RCTs included in the meta-analysis.

First author (year)	Identifier/ registration	Study design	Study phase	Cancer	Treatment group (No.)	Control group (No.)
Bardia (7)	ASCENT/ NCT02574455	RCT	3	metastatic triple-negative breast cancer	sacituzumab govitecan (267)	eribulin, vinorelbine, capecitabine, or gemcitabine (262)
Rugo (15)	TROPiCS-02/ NCT03901339	RCT	3	HR+ HER2- locally recurrent inoperable or metastatic breast cancer	sacituzumab govitecan (272)	eribulin, vinorelbine, capecitabine, or gemcitabine (271)
Xu (19)	EVER-132-002/ NCT04639986	RCT	3	HR+ HER2- metastatic breast cancer	sacituzumab govitecan (166)	eribulin, vinorelbine, capecitabine, or gemcitabine (165)
Paz-Ares (8)	EVOKE-01/ NCT05089734	RCT	3	metastatic non–small cell lung cancer	sacituzumab govitecan (299)	docetaxel (304)
Powles (9)	TROPiCS-04/ NCT04527991	RCT	3	locally advanced unresectable or metastatic urothelial carcinoma	sacituzumab govitecan (355)	paclitaxel, docetaxel, or vinflunine (356)

RCT, randomized controlled trial; No., number; HR, hormone receptor; HER2, human epidermal growth factor receptor 2.

**Table 2:** Summary of main outcomes of the included RCTs.

First author (year)	Comparator	Age (range)	Previous systemic regimens, No., median (range)	Patients (n)	Response RECIST (n)	PFS (mo)	PFS HR (95% CI)	OS (mo)	OS HR (95% CI)
Bardia (7)	SG	54 (27-82)	4 (2-17)	267	CR:10 PR:73 SD:96 PD:65	4.8	0.41 (0.33-0.52)	11.8	0.5 (0.42-0.63)
	Chemotherapy	53 (27-81)	4 (2-14)	262	CR:2 PR:9 SD:71 PD:100	1.7		6.9	
Rugo (15)	SG	57 (49–65)	/	272	CR:2 PR:55 SD:142 PD:58 NE:15	5.5	0·66 (0·53–0·83)	14.4	0.79 (0·65–0·96)
	Chemotherapy	55 (48–63)	/	271	CR:0 PR:38 SD:106 PD:76 NE:51	4.0		11.2	
Xu (19)	SG	53(32-72)	2 (1–4)	166	CR:2 PR:32 SD:93 PD:36 NE:3	4.3	0.67 (0.52–0.87)	21.0	0.64 (0.47–0.88)
	Chemotherapy	51(28-79)	2 (1–4)	165	CR:0 PR:25 SD:82 PD:45 NE:13	4.2		15.3	
Paz-Ares (8)	SG	66 (31-84)	/	299	CR:0 PR:41 SD:161 PD:66 NE:31	4.1	0.92 (0.77-1.11)	11.1	0.84 (0.68-1.04)
	Chemotherapy	64 (32-83)	/	304	CR:3 PR:52 SD:149 PD:64 NE:36	3.9		9.8	
Powles (9)	SG	67 (41-89)	2 (1-7)	355	CR:19 PR:61 SD:151 PD:75 NE:49	4.2	0.86 (0.72-1.03)	10.3	0.86 (0.73-1.02)
	Chemotherapy	68 (30-85)	2 (1-6)	356	CR:9 PR:40 SD:170 PD:77 NE:60	3.6		9.0	

SG, Sacituzumab govitecan; PFS, progression-free survival; OS, overall survival; HR, hazard ratio; CI, confidence interval; n, number; mo, months; CR, complete response; PR, partial response; SD, stable disease; PD, progressive disease; NE, not evaluable.

#### 3.2.2 Study quality and risk of bias

**Figure 2:**
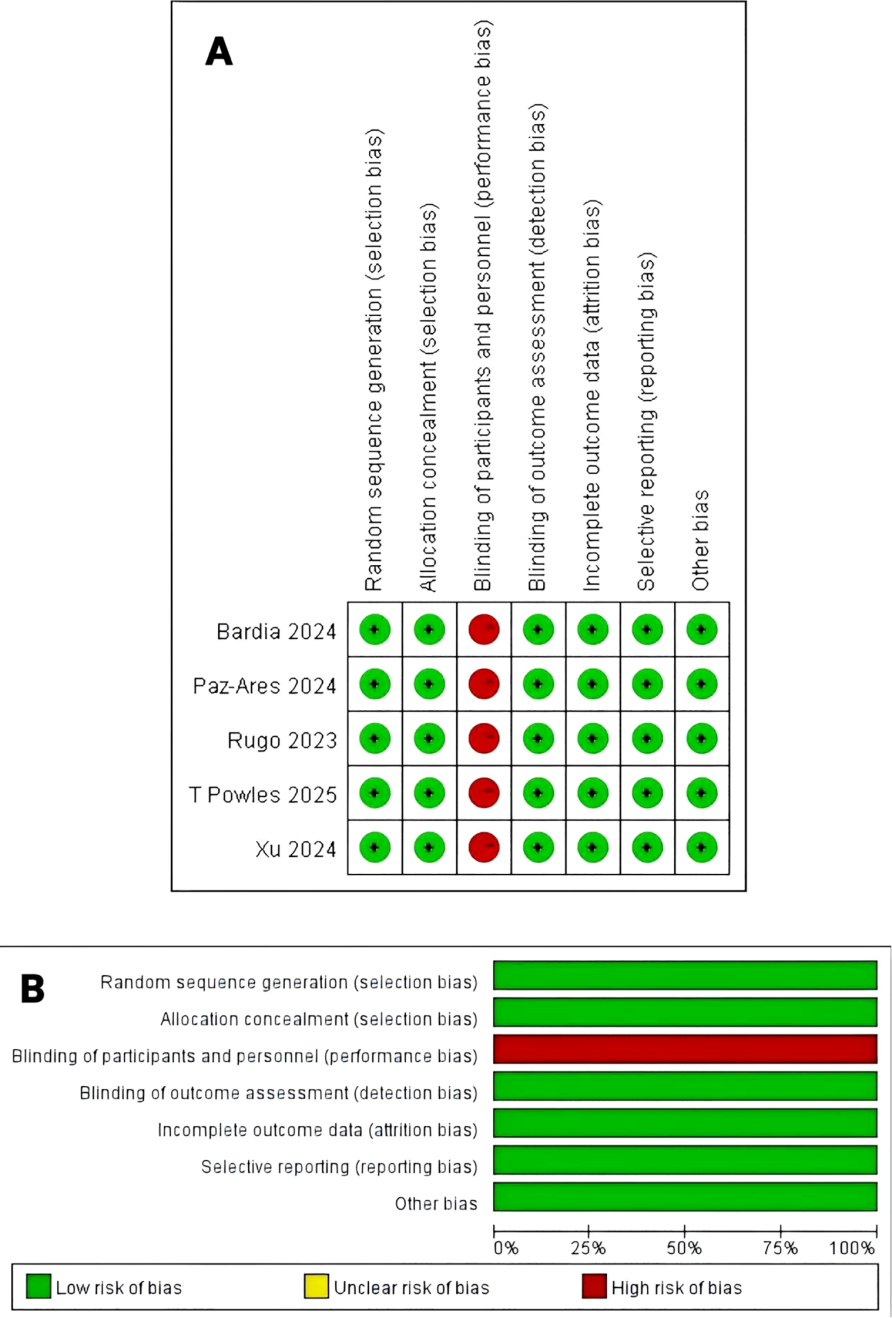
Risk of bias. **(A)** Summary: review authors’ judgments about each risk of bias item for each included RCT. **(B)** Graph: review authors’ judgments about each risk of bias item presented as percentages across all RCTs.

All five RCTs reported outcomes including OS, PFS, ORR, DCR and TEAEs (7–9, 15, 19). Performance bias was the most frequently identified concern. The overall risk of bias assessment is presented in **Figure 2**.

#### 3.2.3 OS and PFS

Each RCT reported HRs and 95% CIs for OS and PFS (7–9, 15, 19). Substantial heterogeneity was identified for both OS (P = 0.001, I^2^ = 78.6%) and PFS (P < 0.001, I^2^ = 88.6%). To investigate potential sources of heterogeneity, subgroup analyses were performed based on tumor type (breast cancer vs. other malignancies), median patient age (≥60 years vs. <60 years), number of prior systemic treatment lines (>2 vs. ≤2), control group treatment modality (physician’s choice vs. docetaxel), and sample size (>540 vs. ≤540). The analysis suggested that heterogeneity in OS was significantly associated with sample size (between-group heterogeneity: P = 0.001), whereas no significant associations were observed for the other variables. In contrast, heterogeneity in PFS appeared to be influenced by tumor type (P = 0.01), median age (P = 0.01), and control group treatment modality (P = 0.044).

Despite the observed heterogeneity, SG demonstrated clear superiority over chemotherapy, significantly improving both OS (RR = 0.720; 95% CI: 0.587–0.882; P = 0.002) and PFS (RR = 0.682; 95% CI: 0.516–0.901; P = 0.007).

**Figure 3:**
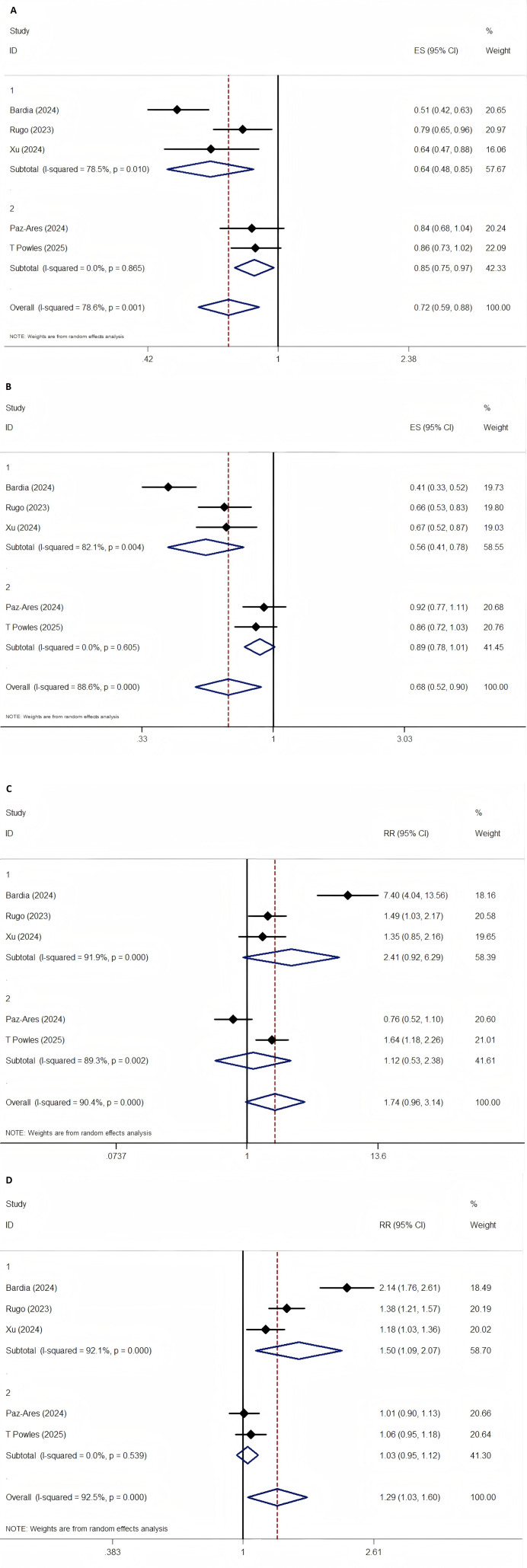
The forest plot comparing of PFS, OS, ORR, DCR between SG and chemotherapy. **(A)** OS, **(B)** PFS, **(C)** ORR, **(D)** DCR.

Subgroup analysis further revealed that patients with BC derived the most substantial benefit from SG, with significantly prolonged OS (RR = 0.637; 95% CI: 0.478–0.849; P = 0.002) and PFS (RR = 0.565; 95% CI: 0.409–0.779; P < 0.001). Pooled survival outcomes are illustrated in **Figures 3A and B**.

#### 3.2.4 ORR and DCR

All five RCTs reported data on ORR and DCR (7–9, 15, 19). Significant heterogeneity was identified for both ORR (P < 0.001, I^2^ = 90.4%) and DCR (P < 0.01, I^2^ = 92.5%). To investigate potential sources of this heterogeneity, subgroup analyses were conducted based on the same five factors used in the analyses of OS and PFS: tumor type, median patient age, number of prior systemic therapy lines, control group treatment modality, and sample size. The findings indicated that heterogeneity in ORR may be attributable to the control group treatment modality (between-group heterogeneity: P = 0.004). In contrast, heterogeneity in DCR appeared to be associated with tumor type (P = 0.019), median age (P = 0.019), number of prior systemic therapies (P = 0.038), and control group treatment modality (P = 0.030).

Although SG did not significantly improve ORR compared to chemotherapy (RR = 1.737; 95% CI: 0.961–3.138; P = 0.067), it was associated with a significantly higher DCR (RR = 1.286; 95% CI: 1.034–1.599; P = 0.024). Forest plots for these outcomes are shown in **Figures 3C, D**.

Subgroup analysis in patients with BC showed no significant improvement in ORR (RR = 2.406; 95% CI: 0.920–6.291; P = 0.073), but a significant increase in DCR (RR = 1.504; 95% CI: 1.095–2.066; P = 0.012).

#### 3.2.5 Treatment-emergent adverse events

Grade ≥3 TEAEs were analyzed to evaluate the safety profile. When all five RCTs were combined, substantial heterogeneity was observed, likely attributable to differences in the control chemotherapy regimens. Specifically, while ASCENT (7), TROPiCS-02 (15), EVER-132–002 (19), and TROPiCS-04 (9) permitted physician’s choice of chemotherapy, EVOKE-01 (8) exclusively used docetaxel. Removing EVOKE-01 from the analysis significantly reduced the heterogeneity of some TEAEs (especially diarrhea and anemia). Meanwhile, sensitivity analyses found that EVOKE-01 has the largest offset, especially in neutropenia and leukopenia groups. Therefore, the pooled safety analysis was based on the remaining four trials.

**Figure 4:**
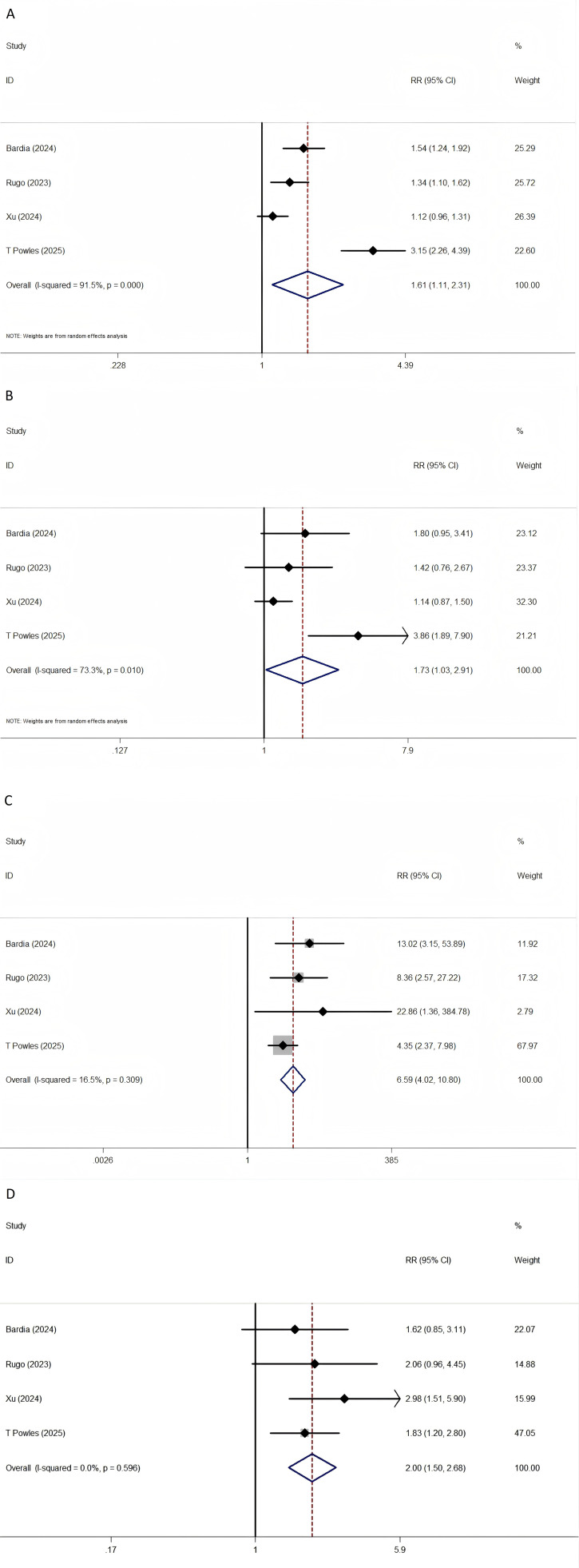
The forest plot comparing of TEAEs between SG and chemotherapy. **(A)** neutropenia, **(B)** leukopenia, **(C)** diarrhea, **(D)** anemia.

SG was associated with a significantly increased risk of neutropenia (RR = 1.606; 95% CI: 1.115–2.314; P = 0.011), leukopenia (RR = 1.731; 95% CI: 1.029–2.912; P = 0.039), diarrhea (RR = 6.592; 95% CI: 4.025–10.797; P < 0.001), and anemia (RR = 2.004; 95% CI: 1.499–2.679; P < 0.001). These results are summarized in **Figure 4**.

Subgroup analysis indicated that patients with UC had higher incidences of neutropenia and leukopenia, while those with BC were more likely to experience diarrhea (**Figure 4**).

#### 3.2.6 Publication bias

**Figure 5:**
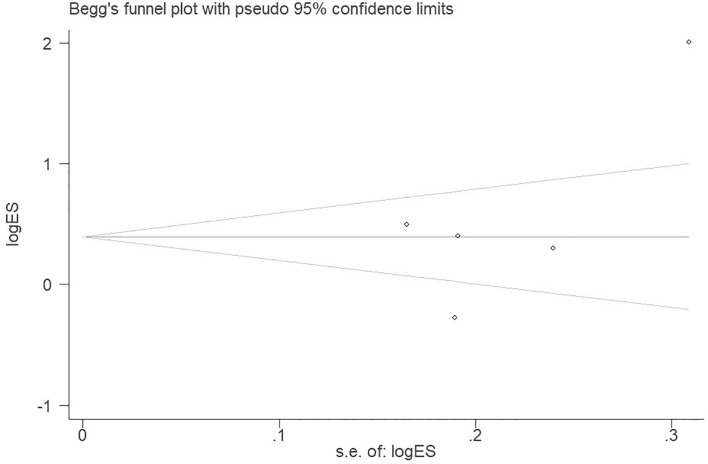
Funnel plot of odds ratio for the included studies.

Publication bias was assessed using Begg’s funnel plots and Egger’s test across the five included studies. The funnel plots demonstrated no apparent asymmetry, and Egger’s test revealed no statistically significant evidence of publication bias (P > 0.05; **Figure 5**).

### 3.3 Single-arm trials

#### 3.3.1 General characteristics

Sixteen single-arm studies (10, 13, 20–26) derived from five clinical trials were included, comprising a total of 769 patients. The IMMU-132–01 basket trial contributed ten subgroups (10, 13, 20), while TROPHY-U-01 (23, 24) and TROPiCS-03 (25, 26) each contributed two. The tumor types included four subgroups of BC (13, 20–22), three of UC (10, 23, 24), two of small cell lung cancer (SCLC) (10, 26), and two of endometrial cancer (10, 25). Additional subgroups from IMMU-132–01 (10) included NSCLC, colorectal, esophageal, pancreatic, and prostate cancers. All studies reported ORR and DCR. Clinical benefit rate (CBR) was available for all studies except ASCENT J02 (22). PFS was reported in 13 studies (10, 13, 20, 23–26), OS in 11 (10, 13, 20, 23, 24, 26), and duration of response (DOR) in eight (10, 13, 20, 23, 24, 26). Key study characteristics are summarized in **Table 3**.

**Table 3:** General characteristics and main outcomes of the included single-arm trials.

First author (year)	Identifier/ registration	Study design	Cancer (number)	Median duration of treatment (mo)	Previous anticancer regimens number (range)	Outcome
Bardia (13) ^a^	IMMU-132-01 Basket Trial/ NCT01631552	1/2	mTNBC (108)	3.7 (range: 0–55.2)	3 (2–10)	The ORR was 33.3%, median DOR was 7.7 months (95% CI: 4.9–10.8), the CBR was 45.4%. Median PFS was 5.5 months (95% CI: 4.1 to 6.3), and OS was 13.0 months (95% CI: 11.2 to 13.7).
Kalinsky (20) ^b^			Breast cancer (HR+ and HER2−/+) (54)	4.6 (range: 0.0–29.4)	/	The ORR was 31.5%, median DOR was 8.7 months (95% CI: 3.7–12.7). Median PFS was 5.5 months (95% CI: 3.6– 7.6), and median OS was 12 months (95% CI: 9.0–18.2).
Bardia (10) ^c^			Urothelial cancer (45)	/	2 (1-6)	The ORR was 28.9%, median DOR was 12.9 months (95% CI: 3.8-22.5), the CBR was 44.4%. Median PFS was 6.8 months (95% CI: 3.6– 9.7), and median OS was 16.8 months (95% CI: 9.0–21.9).
Bardia (10) ^d^			SCLC (62)	/	2 (1-7)	The ORR was 17.7%, the CBR was 24.2%, DOR was 5.7 months (95% CI: 3.6–19.9). Median PFS was 3.7 months (95% CI: 2.1- 4.8), median OS was 7.1 months (95% CI: 5.6-8.1).
Bardia (10) ^e^			NSCLC(54)	/	/	The ORR was 16.7%, the CBR was 24.1%, median DOR was 6.0 (95% CI: 2.5-21.0). Median PFS was 4.4 months (95% CI: 2.5-5.4), and median OS was 7.3 months (95% CI: 5.6-14.6).
Bardia (10) ^f^			Colorectal cancer (31)	/	/	The ORR was 3.2%, the CBR was 19.4%, DOR was 9.8 months (95% CI: NR-NR). Median PFS was 3.9 months (95% CI: 1.9-5.6),and median OS was 14.2 months (95% CI: 6.8-19.1).
Bardia (10) ^g^			Esophageal cancer (19)	/	/	The ORR was 5.3%, the CBR was 21.1%, DOR was 5.4 months (95% CI: NR-NR). Median PFS was 3.4 months (95% CI: 1.9-6.0), median OS was 7.2 months (95% CI: 4.9-14.7).
Bardia (10) ^h^			Endometrial cancer (18)	/	/	The ORR was 22.2%, the CBR was 44.4%. Median PFS was 3.2 months (95% CI: 1.9-9.4), median OS was 11.9 months (95% CI: 4.7-NC).
Bardia (10) ^i^			Pancreatic ductal adenocarcinoma (16)	/	/	The ORR was 0%, the CBR was 0%. Median PFS and OS were 2.0 months (95% CI: 1.1-3.5) and 4.5 months (95% CI: 2.9-7.0).
Bardia (10) ^j^			Castrate-resistant prostate (11)	/	/	The ORR was 9.1%, the CBR was 27.3%. Median PFS and OS were all NP.
Xu (21)	EVER-132-001/ NCT04454437	2	mTNBC (80)	5.59 (95% CI: 5.585–NA)	4 (2-8)	The ORR was 38.8%, the CBR was 43.8%, the median DOR was 5.59 months (95% CI: 5.585–NA). Median PFS was 5.55 months (95% CI: 4.14–NA), and median OS was not evaluable at the data cutoff date.
Naito (22) ^k^	ASCENT J02/NCT05101096	1/2	mTNBC (36)	6.2 (95% CI: 3.1–NR)	/	The confirmed ORR was 25%, median DOR was 6.2 months (95% CI: 3.1–NR). Median PFS was 5.6 months (95% CI: 3.9–NR), median OS was not reached at the time of this analysis.
Tagawa (23) ^l^	TROPHY-U-01/ NCT03547973	2	Urothelial cancer (113)	7.2 (95% CI: 4.7-8.6)	3 (1-8)	The ORR was 27%, the CBR was 37.2%, median DOR was 7.2 months (95% CI: 4.7–8.6). Median PFS and OS were 5.4 months (95% CI: 3.5-7.2) and 10.9 months (95% CI: 9.0-13.8).
Petrylak (24) ^m^			Urothelial cancer (38)	5.6 (95% CI: 2.8-13.3)	2 (1-5)	The ORR was 32%, the CBR was 42%, the median DOR was 5.6 months (95% CI: 2.8-13.3). Median PFS was 5.6 months (95% CI: 4.1-8.3), and median OS was 13.5 months (95% CI: 7.6-15.6).
Santin (25)	TROPiCS-03 (IMMU-132-11)/NCT03964727	2	Endometrial cancer (41)	8.8 (95% CI: 2.8-NE)	3 (1-6)	The ORR was 22%, the CBR was 32%, median DOR was 8.8 months (95% CI: 2.8-NE). Median PFS was 4.8 months (95% CI: 2.8-9.8), the OS data were not mature at the time of data extraction.
Dowlati (26)			Extensive-stage-SCLC (43)	12.3 (range: 8.1–20.1)	/	The ORR was 41.9% (95% CI: 27.0%–57.9%), The median DOR was 4.73 months (95% CI: 3.52–6.70), the DCR was 83.7%, and the CBR was 48.8%. Median PFS and OS were 4.40 (95% CI: 3.81–6.11) and 13.60 (95% CI: 6.57–14.78) months.

mTNBC, metastatic triple-negative breast cancer; HR, hormone receptor; HER2, human epidermal growth factor receptor 2; SCLC, small cell lung cancer; NSCLC non-small cell lung cancer; NA, not available; NE, not estimable; NC, not calculable; NP, not provided; CI, confidence interval; ORR, overall response rate; DCR, disease control rate; PFS, progression-free survival; OS, overall survival; CBR, clinical benefit rate.

^a,b,c,d,e,f,g,h,i,j^ Belong to different cohorts of the same clinical study.

^k^Only the result of the mTNBC cohort in phase 2 were analyzed.

^l,m^ Belong to different cohorts of the same clinical study.

#### 3.3.2 PFS, OS and DOR

**Figure 6:**
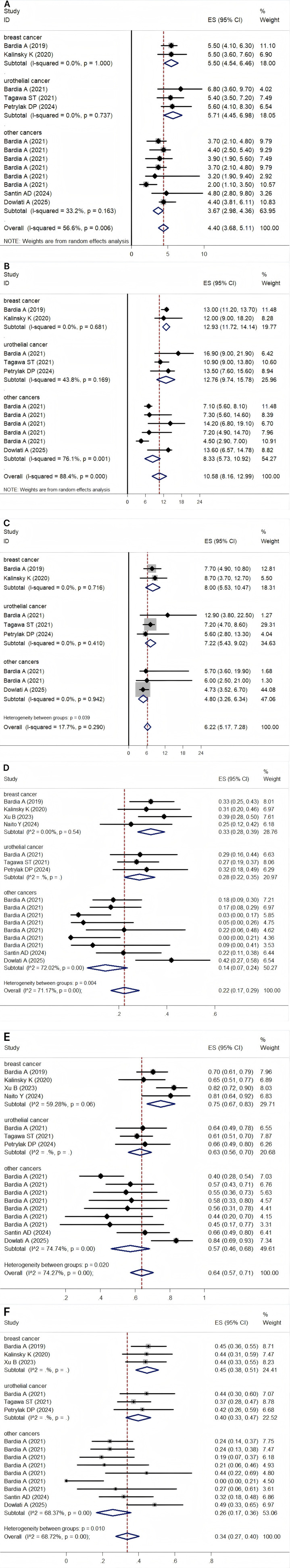
The forest plot of related single-arm trials for treating solid tumors with SG. **(A)** PFS, **(B)** OS, **(C)** DOR, **(D)** ORR, **(E)** DCR, **(F)** CBR.

The pooled median PFS, calculated using a random-effects model (I^2^ = 56.6%, P = 0.006), was 4.40 months (95% CI: 3.68–5.11 months). Subgroup analyses showed median PFS values of 5.50 months (95% CI: 4.54–6.46 months) for BC, 5.71 months (95% CI: 4.45–6.98 months) for UC, and 3.67 months (95% CI: 2.98–4.36 months) for other tumor types. The pooled median OS, also estimated using a random-effects model (I^2^ = 88.4%, P < 0.001), was 10.58 months (95% CI: 8.16–13.00 months). Median OS was 12.93 months (95% CI: 11.72–14.14 months) in the BC subgroup and 12.76 months (95% CI: 9.74–15.78months) in the UC subgroup. DOR was analyzed using a fixed-effects model (I^2^ = 17.7%, P = 0.290), resulting in a pooled median DOR of 6.23 months (95% CI: 5.17–7.28 months). The longest median DOR was observed in the BC subgroup at 8.00 months (95% CI: 5.53–10.47 months). Forest plots for these outcomes are presented in **Figures 6A–C**.

#### 3.3.3 ORR, DCR and CBR

Marked heterogeneity was observed for ORR, DCR, and CBR, as indicated by I^2^ values greater than 50% and Q-test p-values less than 0.01. The ORR ranged from 0.00% to 41.86%, with a pooled estimate of 22% (95% CI: 17–29%). Subgroup analysis revealed the highest ORR in BC at 33% (95% CI: 28–39%), followed by UC at 28% (95% CI: 22–35%), and other tumor types at 14% (95% CI: 7–24%) (**Figure 6D**). The DCR ranged from 40.32% to 83.72%, with a pooled rate of 64% (95% CI: 57–71%). Among subgroups, DCR was highest in BC at 75% (95% CI: 67–83%), followed by UC at 63% (95% CI: 56–70%), and other cancers at 57% (95% CI: 46–68%) (**Figure 6E**). The CBR ranged from 0.00% to 48.84%, with a pooled estimate of 34% (95% CI: 27–40%). The BC subgroup had the highest CBR at 45% (95% CI: 38–51%), followed by UC at 40% (95% CI: 33–47%), and other tumor types at 26% (95% CI: 17–36%) (**Figure 6F**).

## 4 Discussion

ADCs are currently under investigation for their efficacy and safety in the treatment of solid tumors, both as monotherapy and in combination with other therapeutic agents. Among these, SG has shown particular promise, especially in BC and UC, where it has attracted considerable clinical interest (7, 9, 15, 19). Although SG has been evaluated across a broad spectrum of solid malignancies, several recent trials have reported variable outcomes, with some indicating limited therapeutic benefit (8, 9). These heterogeneous findings underscore the need to delineate the tumor types most likely to derive substantial benefit from SG. To address this, we conducted a comprehensive meta-analysis to evaluate the efficacy and safety of SG across multiple solid tumor types.

Our analysis, which included five RCTs comparing SG to standard chemotherapy across various malignancies, demonstrated significant improvements in OS, PFS and DCR. Subgroup analyses revealed that patients with BC derived the greatest clinical benefit, exhibiting both superior short-term response rates and enhanced long-term survival outcomes. In a previous meta-analysis, Qureshi et al. assessed the efficacy of SG in patients with HR+/HER2- advanced BC. Two studies were eligible for inclusion, and the results showed a significant improvement in PFS and DOR, although no significant difference in OS was observed between the SG and control groups (12). To further explore the therapeutic potential of SG across different malignancies, Sultana et al. evaluated its clinical efficacy in a range of solid tumors, including BC, UC, NSCLC, SCLC, colorectal cancer, pancreatic ductal adenocarcinoma, esophageal cancer, castration-resistant prostate cancer, endometrial cancer, and other epithelial malignancies. The pooled median PFS and OS were 4.9 months (95% CI: 4.0–5.8 months) and 9.6 months (95% CI: 7.6–11.6 months), respectively (27). However, only two of the ten studies included in their analysis were RCTs; the remainder comprised cohort studies and one real-world evidence report. While our meta-analysis incorporated only five studies, all were phase 3 RCTs with large sample size, enhancing the robustness and generalizability of our findings. This provides a higher level of evidence and may offer more definitive guidance for clinical decision-making regarding the use of SG in solid tumors.

Given the limited number of RCTs, relevant single-arm studies were also included to supplement the analysis. These studies further supported the observed trends, showing more favorable outcomes with SG in BC and UC compared to other tumor types. However, the inherent limitations of single-arm trials—such as the absence of a control group, small sample sizes, and susceptibility to various confounding factors—reduce their scientific rigor and confirmatory value relative to RCTs.

Although ADCs are generally perceived as less toxic than traditional chemotherapy, our meta-analysis revealed a higher incidence of AEs associated with SG. The most frequently reported AEs included neutropenia, leukopenia, diarrhea, and anemia. Subgroup analyses suggested differential toxicity profiles among tumor types. In particular, patients with BC appeared more prone to developing diarrhea, whereas those with UC exhibited a higher risk of neutropenia and lymphopenia, warranting heightened clinical vigilance in these populations. Notably, the TROPiCS-04 trial (9) reported 25 deaths in the SG treatment group, 16 of which were infection-related and occurred in the setting of neutropenia. Fourteen of these deaths occurred within the first month of treatment. All 16 patients had multiple risk factors that would typically warrant primary prophylaxis with granulocyte colony-stimulating factor (G-CSF), including age ≥65 years, prior anticancer therapies, and the presence of multiple comorbidities. Despite this, only two of these patients received primary G-CSF prophylaxis, while nine received it as treatment after neutropenia had developed. These findings underscore the critical importance of early neutrophil monitoring and proactive management of neutropenia during SG therapy, particularly in patients with identifiable risk factors. Efforts have also been made to identify high-risk populations for treatment-related AEs. Given the central role of uridine diphosphate-glucuronosyltransferase family 1 member A1 (UGT1A1) in the metabolism of SN-38, the active metabolite of SG, this enzyme has been the focus of considerable investigation. Several studies have reported that patients homozygous for the UGT1A1*28* allele experience a higher incidence of severe neutropenia compared to non-carriers (10, 13). The ASCENT trial further suggested the relevance of genotype-directed dosing, as treatment*-*related AEs were more frequent in patients with the UGT1A128/*28 genotype compared to heterozygous or wild-type individuals (7), indicating that UGT1A1 genotyping may help predict SG-related toxicities. However, a meta-analysis evaluating the association between SG toxicity and UGT1A1 genotype across 11 clinical studies involving various solid tumors found no significant difference in AEs between genotypes. This result may be attributable to the lack of genotype data in more than 50% of the included studies (28). Therefore, larger, well-designed, genotype-informed studies are necessary to clarify the clinical utility of UGT1A1 testing in predicting SG-related toxicities.

Given the limitations of SG monotherapy in certain tumor types, combination strategies are currently under investigation. These include regimens integrating SG with immune checkpoint inhibitors, other ADCs (28), and radiotherapy (29). The results of these ongoing large-scale trials are eagerly anticipated and may provide new avenues to enhance therapeutic efficacy and broaden the clinical applicability of SG.

## 5 Conclusion

This systematic review demonstrates that SG provides superior OS, PFS, and DCR compared to standard chemotherapy in patients with solid tumors. To address the limited availability of RCT data, relevant single-arm studies were also included to enhance clinical insight. The findings suggest that patients with BC and UC derive the most substantial benefit from SG treatment. However, the methodological limitations of single-arm trials—such as lack of a control group and potential bias—must be carefully considered when interpreting these results. Moreover, SG is associated with a higher incidence of TEAEs, highlighting the importance of vigilant toxicity monitoring and management. Further research, particularly well-designed, large-scale RCTs, is essential to more definitively establish the clinical efficacy and safety profile of SG across diverse tumor types.

## Data Availability

The datasets presented in this study can be found in online repositories. The names of the repository/repositories and accession number(s) can be found in the article/**Supplementary Material**.
